# Aligning mission to digital health strategy in academic medical centers

**DOI:** 10.1038/s41746-022-00608-7

**Published:** 2022-06-02

**Authors:** Adam B. Cohen, Lisa Stump, Harlan M. Krumholz, Margaret Cartiera, Sanchita Jain, L. Scott Sussman, Allen Hsiao, Walter Lindop, Anita Kuo Ying, Rebecca L. Kaul, Thomas J. Balcezak, Welela Tereffe, Matthew Comerford, Daniel Jacoby, Neema Navai

**Affiliations:** 1 Yale New Haven Health, 20 York Street, New Haven, CT 06510 USA; 2 The University of Texas MD Anderson Cancer Center, 1515 Holcombe Blvd., Houston, TX 77030 USA

**Keywords:** Health services, Medical research

## Abstract

The strategies of academic medical centers arise from core values and missions that aim to provide unmatched clinical care, patient experience, research, education, and training. These missions drive nearly all activities. They should also drive digital health activities – and particularly now given the rapid adoption of digital health, marking one of the great transformations of healthcare; increasing pressures on health systems to provide more cost-effective care; the pandemic-accelerated funding and rise of well-funded new entrants and technology giants that provide more convenient forms of care; and a more favorable regulatory and reimbursement landscape to incorporate digital health approaches. As academic medical centers emerge from a pandemic-related reactionary digital health posture, where pressures to adopt more digital health technologies mount, a broad digital health realignment that leverages the strengths of such centers is required to accomplish their missions.

## Introduction

Can a large, complex health system, such as an academic medical center, lead in healthcare if it delivers unrivaled in-person care but only substandard digital health offerings?

The strategies of academic medical centers arise from core values and missions that aim to provide unmatched clinical care, patient experience, research, education, and training. These missions drive nearly all activities with one common, glaring omission – digital health. As academic health institutions struggle to emerge from a pandemic-related reactionary digital health posture, a broad digital health realignment is required to accomplish their missions.

Recently in npj Digital Medicine, Marwaha and colleagues offer a useful approach to implementing digital health products and services within large, complex medical systems^1^. Their paper provides practical guidance, containing nine assessment categories to help health systems successfully select and implement digital health solutions toward elevating the pursuit of excellence in care.

We aim to address the period *before* such technologies are evaluated and implemented. We focus on academic medical centers, although many of the core tenants apply more broadly. In this contemplation period, an academic medical center must first ask “what digital health solutions will advance institutional excellence as an essential facilitator of the mission, and align with the core values and constituents, including the workforce and patients?” Any alternative, non-mission grounded pathways will create disjointed, fragmented, and ineffective digital health strategies, worsening efficiencies, disrupting care, and compromise the missions.

To deliver the highest level of care and successfully integrate digital health tools into operations, digital health strategy should address one or more parts of the following: unsurpassed clinical care and experiences, advancements to the state of medicine and science, and unrivaled clinical education and training. Collectively, each component contributes to a “Learning Digital Health System” that advances the more holistic “Learning Health System”^2–5^. Academic medical centers, have an obligation to rigorously study the implications and impact of digital health. The very nature of digital health solutions enables data collection quality and volumes from environments unimaginable with traditional care methods. In practice, a Learning Digital Health System should power research, care improvement, and operational insights.

We propose a proactive strategic approach, informed by institutional core values, including five key elements (Table 1): (1) Best-in class: Best-in-class care is often set by academic medical centers and the experts within their walls, adopting only care products and services proven to be most effective, including digital health ones. (2) Embrace the edge: A best-in-class approach can only occur if the institution deeply understands the cutting edge of technology and innovation and what solutions should be embraced. (3) Training and education: The trainees and students learning medicine within these institutions not only provide patient care, which increasingly requires digital health approaches, but also represent the next generation of leaders who will provide and define best-in-class care. They must also learn and embrace digital health technologies. (4) Data collection: Digital health approaches empower institutions to gather data – many of the technologies that can improve clinical outcomes can also capture them. This new approach to data collection empowers clinical research, implementation research, and quality improvement. (5) Research and reports: The digital health industry outside academic medical centers has rapidly outpaced academia by collecting new types of health data in new settings across large populations – academic health centers must adopt this approach while leveraging their research and quality improvement enterprises to make the best use of these data in research and reporting.

**Table 1:** Key components of a mission-aligned digital health strategy for academic health centers, which span clinical, patient experience, research, education, and training missions.

Elements	Core tenants of each component
Best-in-class care	• Evidence-based, high value solutions
	• Ongoing investigations of these solutions
	• Incorporation of institutional clinical expertise
	• Capture and utilization of actionable health data
	• Accessible, usable, and useful solutions
	• Tailors to social determinants or drivers of health
	• Maximizes health equity
Embrace of cutting-edge technologies and human-centered experiences	• Deep understanding of the technology landscape
	• Avoidance of inappropriate use
	• Moving beyond the status quo for clinical and administrative operations
	• Maximizes human-centered experiences
	• Ongoing research-investigations of impact
Training and education	• Digital health and health data literacy
	• Specific training for varied digital health tools
Data collection	• Empower research
	• Empower quality improvement
	• Empower Learning Health Systems
	• Ensure patient privacy, data security, and safe data sharing
Research and reports	• Expansion of the evidence base
	• Appropriate advancement or discontinuation of solutions

We also propose that the time to leverage digital health to advance the missions of academic health systems is now because:

- Large health systems, including academic medical centers have had tremendous experience with various facets of digital health during the current Public Health Emergency, which represents a pandemic silver lining, providing a generational opportunity and foundation from which such centers can continue to transform care and engage patients.
- Patients and providers have increasingly embraced digital health – and already had long adopted digitization in varied other aspects of their lives.
- Academic health system leaders, too, may increasingly embrace digital health solutions as they can represent more efficient and higher value models of care, particularly since the pandemic exacerbated the cost of health system operations through challenges related to the supply chain, labor, and disruptions in traditional revenue sources.
- Many of the enabling technologies have matured beyond conceptual prototypes and are ready for real-world use.
- New tools facilitate the integration of such technologies into the electronic health record (EHR).
- The pandemic-accelerated the race and rise of competitors, many of which include non-traditional health providers, including large retail or technology companies, which are rapidly addressing the demand for more convenient forms of care.
- The regulatory and reimbursement landscape is evolving to become more favorable to incorporating digital health solutions.

Pandemic-related digital health efforts by academic health institutions may have understandably lacked orderly, methodical rollouts. However, haphazard expansion of digital health among these institutions runs the risk of conflicting with core missions and degrading the institutions’ limited resources. To better frame the components of a cohesive and strategic approach to digital health, we first describe the arc of digital health in these medical systems, which starts before and continues through the pandemic.

## Epoch I: Digital health in academic health institutions before COVID-19

In the decade leading up to the COVID-19 pandemic, digital health-focused companies multiplied to the thousands, expanded in the diversity and purpose of offerings, and garnered billions in investment^6^. Most companies focused on disease management instead of prevention or disease detection – telemedicine companies dominated by volume, activity, and amount of investment, as compared to companies in other categories like remote patient monitoring (RPM)^6^. A significant portion of digital health-focused companies centered on direct-to-consumer digital health, which included wearables, virtual reality products, and telemedicine^7^. In addition, large technology and media companies without a health focus also invested heavily in physical digital health products such as wearables and biosensors.

Although many large medical centers began to invest in digital health companies, and create “innovation hubs” to co-develop, invest in, or spin-out companies, few scaled digital health experiences and expertise across clinical operations^8–11^. In the pre-COVID-19 period, clinical adoption was primarily driven by financially incentivizing models. Traditional telemedicine, for example, potentially reduced the use of high-cost in-person resources, and hospital admissions and readmissions for populations with heart failure and chronic obstructive pulmonary disease^12–15^. Such savings were relevant with capitated payer models, while real-world savings in fee-for-service settings have been less clear and are being investigated^16^. Additionally, many large medical centers created ambulatory virtual visit programs or hub-and-spoke relationships where smaller, community spoke hospitals compensated the larger medical system hub hospital for tele-consultation services.

Large medical centers that incorporated digital health solutions into clinical operations mainly focused on telemedicine, and to a lesser extent, RPM^17^. Before the pandemic, most leading telemedicine programs produced small absolute telemedicine visit volumes compared to traditional in-person encounters, which generally mirrored national telemedicine trends^18^. Select large integrated health systems, such as Kaiser Permanente, however, reported using telehealth for over half of its 2015 visit volume^19,20^. Ochsner Health launched RPM for hypertension in 2015 and diabetes in 2017, focusing on a platform that integrated data directly into the EHR^21^. The goal was to gather more information and engage patients between clinical encounters to improve outcomes^21^. While the Centers for Medicare & Medicaid Services (CMS) introduced RPM billing codes in 2018 and expanded them in 2019, these changes alone were insufficient for many institutions to launch large-scale RPM programs^22,23^.

Of the academic and large health systems that offered telemedicine, many such offerings were through contractual relationships with third-party providers to supplement urgent care. Telemedicine was largely not viewed as a means of delivering care to physicians’ existing patients, but rather as a separate and parallel care modality. A variety of specific clinical populations, however, benefited from telemedicine, such as for rapid thrombolytic evaluation in stroke, routine evaluation in Parkinson disease, amyotrophic lateral sclerosis patients who have difficulty traveling to clinic, and immunocompromised patients requiring evaluations after organ transplant, the latter of which was an early use of telemedicine at one of our centers^24–26^.

Among the emerging set of digital health direct-to-consumer offerings, most had not been integrated into clinical practice and thus, had not been a mainstay of the practice of disease monitoring, surveillance, diagnosis, or treatment. Despite the proliferation of technologies that enabled patient-generated health data, the incorporation of these data into care practices was uncertain, as was their impact on care^27^. A variety of institutions nevertheless brought these data into practice by adopting technology platforms that integrated EHRs and wearable health technologies producing patient-generated health data^28^.

Not for lack of ambition or ingenuity, digital health products and services had mostly languished at most academic health institutions^17,18^. When present, these offerings were typically “proofs of concept,” siloed, or executed in limited capacity, often as part of physician scientists’ clinical research. A seemingly innumerable list of regulatory, legal, payor and technical hurdles stymied larger scale efforts^29^. Institutions wrestled with integration into the EHR, use and value of data, impact on clinical workflow, and outcome metrics. In telemedicine, for example, there were payer limitations on “originating sites,” structural limitations against audio-only interactions, severely truncated visit type allowances, and a lack of payer parity when comparing digital (e.g. telemedicine virtual visits) and conventional offerings. Despite more than a decade of intense digital health industry growth, investment, and advocacy, these limitations remained largely fixed until the early months of 2020.

## Epoch II: Digital health in academic health institutions during the current Public Health Emergency

The Public Health Emergency around COVID-19 changed everything^30^. It required a rapid reactionary response from medical centers due to existential threats of three interconnected priorities: patient health, patient engagement, and enterprise economics. Health systems rapidly deployed and continue to expand large-scale connected health solutions. These solutions mainly constituted direct-to-patient telemedicine offerings and became the only pathway to patient care with safe social distancing. Telemedicine enabled institutional economic viability and care continuity.

In 2019, for example, Yale New Haven Health completed 316 ambulatory video visits, but in the face of the pandemic grew to over half a million in 2020. Massive growth was also seen at centers across the nation, including the University of Pennsylvania, Mt. Sinai, University of Pittsburgh Medical Center, Thomas Jefferson University Hospital, and Stanford, among many others^31^. In addition to large health care enterprises, such growth occurred at tertiary specialty centers such as The University of Texas MD Anderson Cancer Center, which now maintains a telemedicine-based encounter volume that represents 20% of all patient encounters.

Some centers, including our own, also launched inpatient telemedicine operations to reduce in-person interactions and the corresponding personal protective equipment use –spanning many contexts including clinical consultation, family meetings, nursing communication, patient monitoring, and education^32,33^.

RPM also expanded and included COVID-19-specific home-based monitoring such as text message check-ins, temperature monitoring, and oxygen saturation assessments, digitally transmitted to clinical monitoring centers^34,35^. RPM programs at institutions such as Mayo Clinic, Intermountain, University of Colorado, and Brigham and Women’s Hospital also expanded^36–39^. Each followed a similar model with centrally monitored patient-reported data. Some expansion efforts in virtual care combined RPM with virtual visits, virtual rehabilitation, and patient coaching.

CMS’s November 2020 amplification of the “Hospital Without Walls” program laid the groundwork for health systems to develop or expand connected care, including “hospital at home” ^40^. Once granted a waiver, systems could bill Medicare and receive inpatient Diagnosis Related Groups (DRG) payments for services rendered in patients’ homes. Many systems stood up or significantly expanded home hospital care for patients to preserve inpatient bed capacity for those patients who required it, regardless of COVID-19 status^41^.

Since care such as infusion therapy and procedures still required in-person visits, various tactics focused on automating and streamlining the pre-visit period. Approaches such as contactless arrival and check-in aimed to reduce the demand on human resources and the transmission of COVID-19. These approaches included digital scheduling, patient survey, and patient intake, leveraging mobile phone functionality and short message service (SMS) text-based communications technologies.

Governmental and payer responses enabled these clinical tele-operations and digital health responses. Notable actions included payer parity with in-person encounters, removal of CMS “originating site” requirements allowing all beneficiaries to receive telemedicine services, relaxation of telemedicine methods by the Health & Human Services Office of Civil Rights allowing HIPAA-noncompliant private communications technologies, and relaxation of state medical board telemedicine requirements^42^. The longevity of these responses is not yet established as some rollbacks have begun.

The pandemic also brought massive increases in activity and funding in the digital health industry – year-over-year doubling of funding since 2019, with tremendous activity in digital health-enabled approaches to mental health care and other chronic conditions such as diabetes mellitus and common musculoskeletal conditions; nearly 90 “megadeals” (companies raising more than $100 M in a fundraising round); four of the five largest digital health deals in over a decade; investments in infrastructure and interoperability solutions that enable incorporation of the digital health solutions into the existing health system infrastructure; and accelerated adoption of digital health tools to generate real-world data and evidence and perform decentralized trials by the pharmaceutical industry^43^.

## Epoch III: Digital health in academic health institutions looking forward

The current state provides a generational opportunity for healthcare enterprises to transform patient care and engagement: Academic centers can leverage their experience, ability, new infrastructure, and determination to operate large-scale digital health operations that began or accelerated with COVID-19. Further, and equally important, the approach must shift from reactive to proactive and from crisis response to strategic. As with the approach to other health institution operations, the mere implementation of digital health solutions into operations does not necessarily mean they enable best-in-class care, patient engagement, research, education, or training. Digital health strategy should stem from the underlying mission with the greatest emphasis on clinical needs and outcomes.

A proactive strategic approach runs counter to the opportunistic and reactionary postures to digital health experienced by many academic medical centers before and since the ongoing Public Health Emergency. Academic medical centers of the future should anticipate needs and proactively invest in mission-aligned digital tools that provide unmatched clinical care, patient experience, research, education, and training at their institution.

We believe the path for health institutions that haphazardly expand their digital health portfolio will run afoul. In contrast to past efforts centered on conventional care, many academic health institutions now embrace non-conventional digital health-enabled care including various offerings, such as video-based telemedicine visits and remote patient monitoring^44^. Unbridled adoption could easily lead to mission deviation, whereby instead of desirable “disruptive innovation” improving efficiency and effectiveness, implementation of “innovative” technologies simply disrupt care, add cost, and produce frustration. Unbridled adoption could also turn institutions into commodities instead of industry partners or customers, whereby the health systems lose the experts’ voice that should guide excellent care, or lose control of data, which would interfere with the research mission.

First, mission-driven digital health-enabled care must provide best-in class care. At the most basic level, this care should be evidence-based and the resultant outcomes measured. Although the evidence supporting the impact of digital health solutions is growing, it remains limited^45,46^. Academic healthcare institutions should champion evidence-backed digital health solutions that address high-priority clinical needs while also promoting higher care value through better outcomes at lower costs. Such institutions, partnering with peer institutions, are also uniquely positioned to identify and study candidate digital health solutions given their large patient volumes, and wealth of experts across primary and specialty services.

When assessing these solutions, implementation teams and investigators should avoid expecting generic tools, like telemedicine-based video visits, will produce universal gains in outcomes or cost reduction – just as “clinic visits appointments” or “surgery” produce differential results based on the populations, conditions, and settings in which they are applied.

These institutions, which may provide primary and general care to large local populations, and tertiary level care across a much larger geographic region, often must tailor solutions to these two prongs of care with consideration of patients’ social drivers of health. Further, the potential solutions must meet key tenants of adoption, many of which have been outlined by Marwaha et al and others^1,47,48^. Attention to these tenants ensures patients can access, understand, use, and benefit from the technologies, including marginalized, lower socioeconomic, or older populations.

Leading solutions will have been directly shown to effectively address high-impact patient needs by rigorous clinical testing. Alternatively, if the digital health solution has yet to be studied, its approach has been backed by sound evidence. Examples of evidence-backed approaches that could have digital health approaches include monitoring seizure frequency in epilepsy^49^, assessment of mood disorders in Parkinson disease^50^, and patient-reported outcomes in heart failure^49–52^. All of these examples also represent the collection of health data that may directly lead to changes in care, which affect clinical outcomes. Digital health solutions need not exceed the comparable in-person or traditional care options, but rather produce at least comparable outcomes while improving convenience, access, patient satisfaction, or cost. For example, a televisit is almost always superior to the scheduled in-person visit that never occurs due to transportation, child-care, or other access issues.

Second, although academic centers should not implement technology for technology’s sake, they should become deeply familiar with the emerging technology and innovation landscape. Only through intimate knowledge, exploration, and use of cutting-edge solutions can their potential be embraced (or dismissed) and realized. And only those who realize the potential of cutting-edge solutions provide best-in-class care. Many centers already have innovation hubs or centers that house such expertise. Such hubs should be explicitly connected to the medical and operational arms of the organizations to best translate knowledge of digital health into practice.

This embrace will enable such institutions to pioneer cutting-edge digital health products, services, and approaches to advance care and mission. It requires a forward-leaning, risk-taking organization that can rapidly deploy and assess a solution’s impact while improving the solution with agility or “failing fast.” The measurement and assessment of deployed digital health technologies should be enabled by the organization’s research and quality improvement arms.

As consumerism across industries creates choice, personalization, and empowerment, patients expect their healthcare experiences to at least match experiences elsewhere. Digital health tools should engage patients through a convenient connected experience that integrates into their lives outside healthcare. This does not imply a “one vendor” or “one technology” approach, which runs counter to innovation. Instead, a systemic approach with deliberate interoperability at data, technology, and patient and provider experience layers.

Various health systems, such as Ochsner Health, Nemours Children’s Health, the Mayo Clinic, the Cleveland Clinic and UPMC, have led large-scale successful telehealth implementations and embrace advanced approaches likely to improve care^21^. Ochsner Health, for example, has scaled its RPM to a nationwide program, covering various clinical conditions, offering health coaching, pharmacist support, and automated technical assistance^21^.

Best-in-class offerings will increasingly include sophisticated technological features and experiences beyond those that exist with most current digital health offerings – and academic centers should be carving this path.

**Fig. 1:**
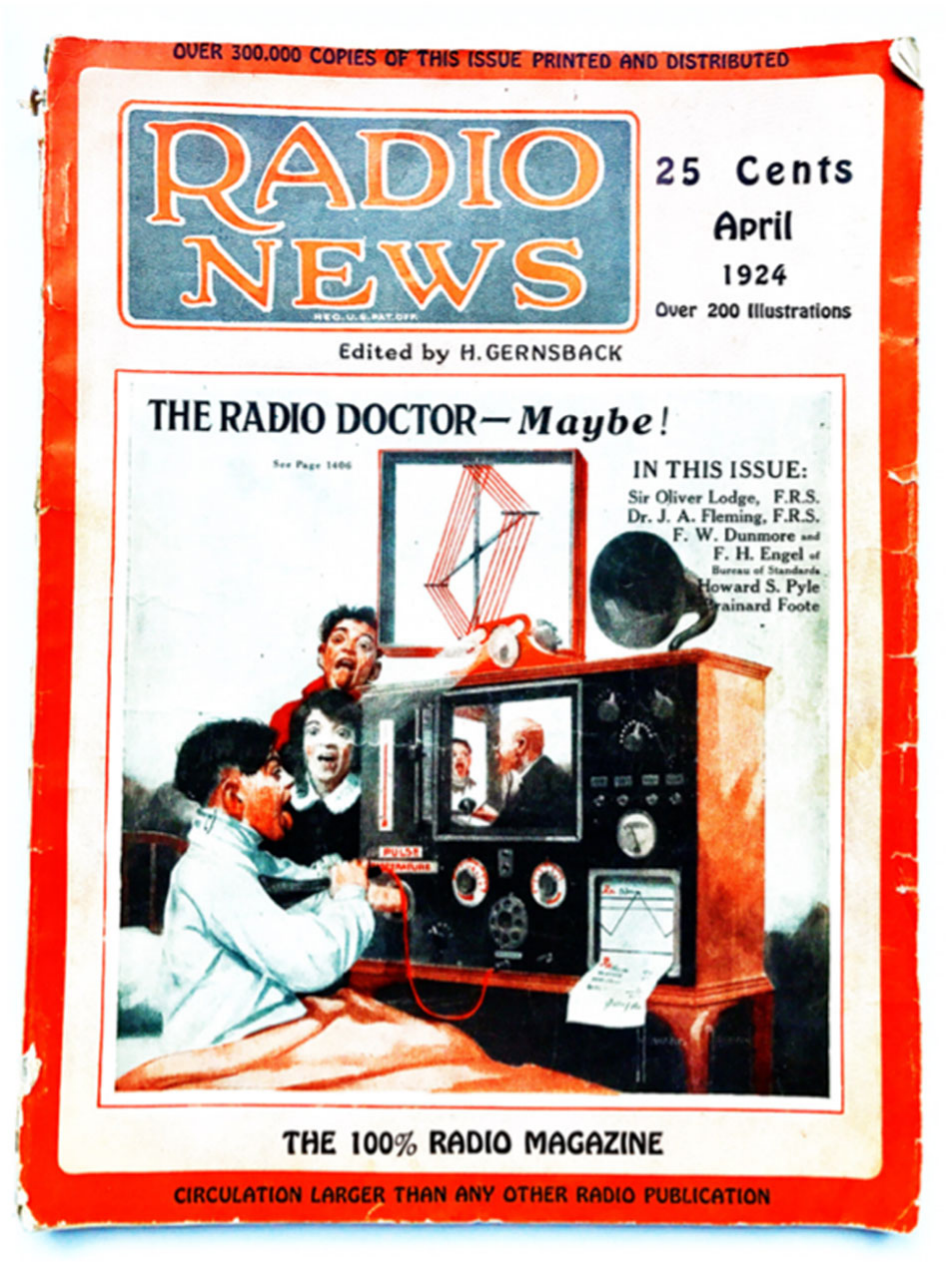
Early telemedicine concept. This is an early conception of telemedicine from Radio News, April 1924. (Radio News is a defunct American magazine, 1919–1971, of which the copyright has expired).

Consider standard telemedicine today, which chiefly entails a simple audio-video connection. The concept was first envisioned on the cover of Radio News in 1924 (Fig. 1)^53^. Also consider most RPM approaches, which largely revolve around the monitoring vital signs and biometric data such as weight.

For how long will these approaches be best-in-class? Expert surveillance and management of cardiology, oncology, and neurology patients at home or on the wards of academic medical centers, for example, often entails much more than a teleconference and the collection of vital signs and biometric data, all of which are useful and necessary but none of which are typically sufficient to provide best-in-class care.

The next phase of such care allows the collection of health data between traditional ambulatory encounters and provides insights into patients’ varied environments and living conditions. Impactful digital health solutions that enable such data exchange will lead to changes in management, provide useful patient insights, and begin to augment or even replace traditional history taking. These technologies should enable the collection of “digital outcome measures,” the “digital examination,” and even the “digital history”^54,55^. Examples of such technologies enable clinically-relevant data collection targeting specific diseases like hypertension, inflammatory bowel disease, COPD, and CHF^54,56–58^.

Beyond basic audio-video televisits, there exist various advanced telemedicine techniques that exploit the digital interface. For example, asynchronous and real-time ophthalmic assessment has enabled providers to successfully capture and assess fundus imagery for triage and disease screening and management^59^. Other remote ophthalmic assessment approaches, such as digital home visual acuity and Amsler grid testing, may be equal or superior to the comparable traditional means^59,60^. These methods augment the monitoring capabilities of ophthalmic experts. They also could enable mass surveillance programs for large primary care populations for which the ophthalmic person-power does not exist to provide in-person assessments. Given the importance of image-based diagnosis in ophthalmology, and existing advanced AI capabilities that produce expert-level image classification, various AI-powered ophthalmology solutions are ready for clinical implementation, such as AI-based image-capture and classification solutions for diabetic retinopathy and macular degeneration^61^.

To advance expert care anytime, anywhere, the system in which patients, providers, and other healthcare staff work must also be as efficient as possible. Efficiency may be increased by tools that allow patients to meaningfully engage with their own health and healthcare data, such as self-scheduling, educational tools, “symptoms checkers,” and chatbots for education, triage, and clinical query. Digital health tools for diagnosis, monitoring, or therapy can be integrated into provider workflow when clinicians can easily “prescribe” them. To realize this, digital formularies will be required for efficient workflow^62^. A multi-health system company collaborative called Graphite Health aims to provide such a formulary, while also promoting a development environment and marketplace that offers digital health products interoperable across different health systems^63^.

Efficiency may also be furthered by novel infrastructure and processes that automate administrative and care processes. Examples include automation of resource-intensive manual tasks like prior authorization, administrative referral communications for potential transplant patients, patient ambient environmental monitoring technology to prevent falls, and resource and bed capacity monitoring^64^.

Third, just as trainees flock to academic medical institutions to learn the latest and most effective methods in diagnosis, procedures, and surgery, such centers should be the beacons of learning to use emerging digital health methods^65,66^. This includes practical training for all types of health providers on digital health literacy, informatics, virtual visits, tele-consultations, RPM, “digital examinations,” and artificial intelligence-assisted and virtual-augmented reality-enabled analysis, interpretation, recommendation, and visualization tools^55^. Just as academic residency and fellowship programs equip budding physicians with the clinical experience and evidence-based medical training and surgical techniques, such programs should provide similarly robust exposure and experiences with basic and advanced digital health tools. Each clinical area or specialty should house digital health expertise, coordinated with internal innovation and digital health hubs, whereby some of these experts represent a new type of clinical educator. Also, many of the tools themselves provide unique educational opportunities, improve access to new types of knowledge, and expand access to educational opportunities to students and trainees without previous access.

Fourth, the data flowing from digital health-powered care should be consistently collected to monitor clinical outcomes and ensure high care quality care. This will serve the research mission, but should be a pillar of the clinical, training, and educational missions, too. Digital health, by its nature, enables data collection across the care continuum, which represents another reason clinical, research, and quality improvement teams should embrace it. Digital health companies, ranging from remote monitoring platforms to asynchronous telemedicine applications, already collect troves of real-world patient-generated health data in settings outside the walls of traditional care settings. By contrast, academic medical centers largely do not. They lack these data and thus, cannot achieve or provide such levels of patient access once they leave the clinic or hospital. This massive asymmetry of information and patient access greatly differentiates the two current states of academic medical centers and industry. Yet, academia is best poised not only to define the most useful data to collect but also how to study it. We believe the most productive path will be through intelligent partnerships between academic and industry that align with each of their missions.

Thus, well beyond what was first envisioned with EHRs, digital health solutions make Learning Digital Health Systems possible whereby patient-generated health data and provider-generated healthcare data are continuously captured to augment research, improve future care, and enable precision or personalized medicine^2^. To collect such data, academic medical centers must develop or adopt new approaches to data collection that allow organized comingling of traditional data from the EHR, for example, with new data sources such as wearables and remote biosensors. Examples include the Johns Hopkins Precision Medicine Analytics Platform (PMAP), the relationship between Google and Mayo Clinic to build the Mayo Clinic Cloud, and Truveta, a multi-health system company collaborative to aggregate and glean insights from cross-institutional healthcare data^67–69^.

This great wealth of data accumulated within health systems comes with great responsibility. Health systems often serve as custodians of the communities in which they physically exist. They may be the best positioned, however, to protect patients’ privacy and data, which face increasing challenges given opportunities to monetize these data and the technological means to identify patients from seemingly unidentified datasets. Data privacy and security, deeply related to enabling and expanding data sharing and utility, are fast-evolving fields, which also should be shaped by academia – Mayo Clinic, for example, recently led a Series A investment in the company TripleBlind, which is co-developing the technology to facilitate these practices^70^.

Fifth and finally, and related to embracing both impactful and cutting-edge digital health solutions, academic clinical institutions should capitalize on this deluge of data through research and reporting. They should study and report on the efficacy of promising new digital health technologies and approaches, championing the efficacious ones while transparently reporting unsuccessful tools. As noted previously, to be “early adopters” of cutting-edge technologies, the research arms of academic healthcare organizations are well-suited to establish and monitor the impact of digital health technologies.

To do this, researchers must be given easy access to these new data sources. They should be armed with knowledge and resources to garner funding outside traditional sources such as the National Institutes of Health. Also, researchers will benefit from efficient organizational pathways to work with industry, where the bulk of digital health innovation resides. Academic institutions should also utilize digital health tools to foster implementation scientific research, which aims to identify the factors that affect real-world uptake of clinical interventions seemingly efficacious and effective in clinical trials^71,72^.

## Organizational structure

**Fig. 2:**
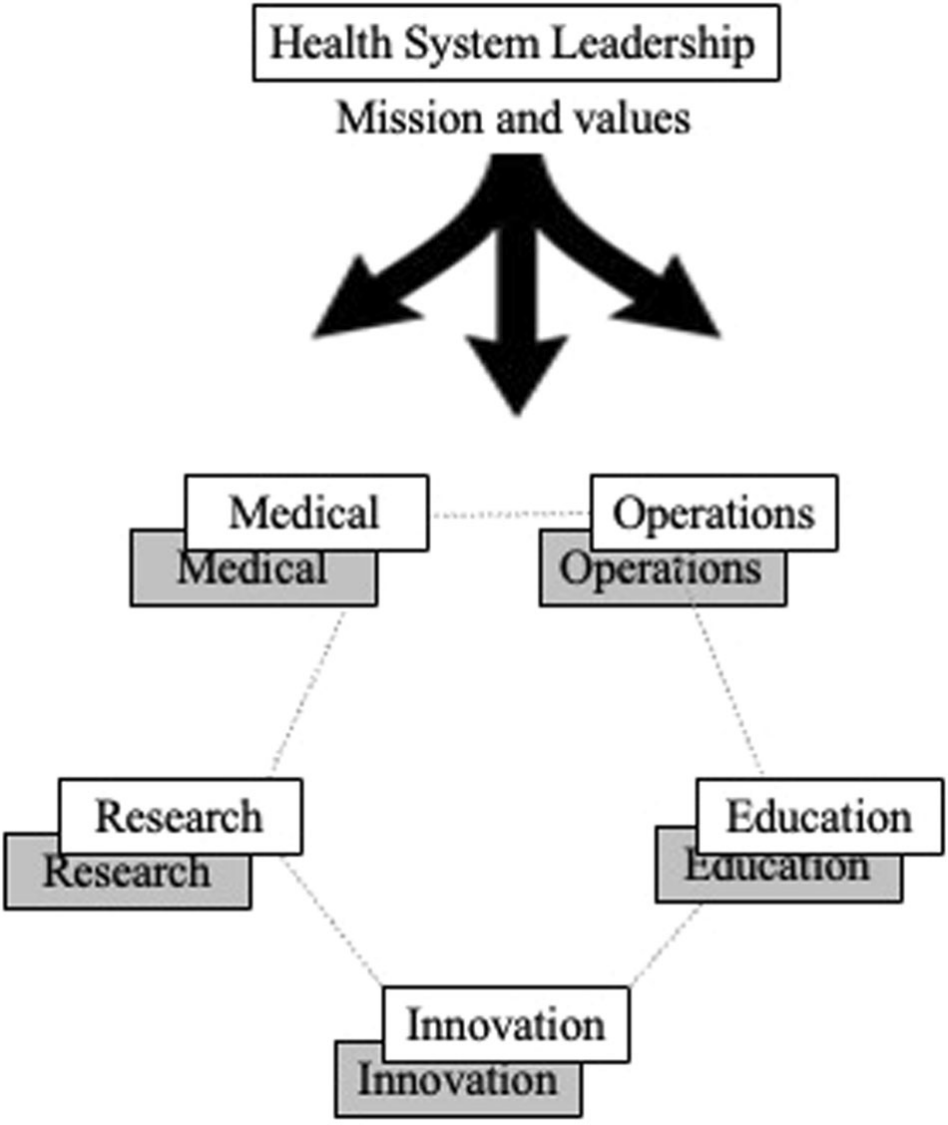
Digital health within academic medical centers. Organizational schema whereby traditional (white boxes) and digital health (gray boxes) components of the organization are delineated but work closely together toward a common mission.

Academic medical centers can integrate digital health into the existing medical, operational, research, education (and training), and innovation components of the organization whereby digital health approaches are considered alongside traditional approaches and not as separate, siloed projects (Fig. 2). Given that digital health requires special expertise, organizations will benefit from dedicated digital health leadership and teams working closely with and not separately from the existing leadership and teams. Advancing digital health also requires close collaboration amongst the leaders of the organization’s strategy, marketing and communications, and information technology arms. These disciplines are organized in a variety of ways across different health systems. The specific organizational structure will differ but all require strong interorganizational collaboration and alignment with common mission, values, priorities, and objectives.

Given the extensive opportunities to advance digital health products and services with inhouse expertise and resources, large medical centers will benefit from an “innovation” arm to the digital health organizational structure^73^. The innovation arm complements the other organizational arms in that they each have separate, albeit interlinked, roles: identify and prioritize the clinical, research, and education and training needs; assess and select technologies and design experiences to address the needs; and implement the solutions. The institutional priorities and core values represent the common thread across each arm, including the focus of innovation – for example, newly developed and adopted solutions must demonstrably improve outcomes in high priority clinical areas.

Although these components will have areas of overlapping interests, the core responsibility of each arm should be clearly defined and operationalized.

## Conclusion

As digital health companies continue to proliferate, expanding their reach and impact, academic medical centers have tremendous pressure to adapt, but also a great opportunity to capitalize on this wave of innovation, enhancing their missions. As these companies increase their ability to directly engage patients by using more on-demand, convenient, and cost-effective methods of care, academic health institutions risk being marginalized to the detriment of many patients who need the expertise and resources held within them. Since many such centers provide primary and general care to large local populations, and expert tertiary level care across a much larger geographic region, they must intentionally adopt digital health solutions that align to these two prongs of care and ensure both are mission-aligned.

Health centers embracing digital health as part of their missions must look past the near-term business case and immediate return-on-investment. They should seek or create digital health solutions that help them maintain and strengthen their place in healthcare, often as the beacons of care. They should work through current regulatory or payer impediments to integrating digital health into practice and also look past them and champion better models^74^.

The necessary ingredients to achieve a strategic mission-driven approach to digital health include prioritized clinical requirements; an effective internal governance structure; in-house expertise on the evolving digital health landscape; and robust, reproducible pathways for piloting, implementing, tracking, and scaling (and retiring) digital health products and services that integrate with and become routine clinical care.
